# Flat dose intravenous immunoglobulin primary infection prophylaxis in multiple myeloma patients on BCMA and GPRC5D bispecific antibody therapy: the Vanderbilt experience

**DOI:** 10.3389/fimmu.2026.1721820

**Published:** 2026-02-19

**Authors:** Naimisha Marneni, Spencer Lessans, Bhagirathbhai Dholaria, Salyka Sengsayadeth, Eden A. Biltibo, Vivek Patel, Shakthi Bhaskar, Reena Jayani, Sanjay Mohan, Ashwin Kishtagari, David Morgan, Olalekan O. Oluwole, Bipin N. Savani, Adetola A. Kassim, Muhamed Baljevic

**Affiliations:** Division of Hematology and Oncology, Department of Medicine, Nashville, TN, United States

**Keywords:** bispecific antibodies (BsAbs), cellular therapies, hypogammaglobinemia, IVIG (intravenous immunoglobulin) administration, multiple myeloma

## Abstract

Bispecific antibodies (BsAbs) can cause profound hypogammaglobulinemia. Conventional infection prophylaxis has utilized weight-based IVIG dosing. However, the use of flat IVIG dosing can significantly reduce IVIG utilization. We evaluated hypogammaglobulinemia and infection risk in relapsed/refractory multiple myeloma (RRMM) patients treated with BsAbs who received flat-dose IVIG for primary infection prophylaxis. Among thirty patients, 73% had ≥1 and 43% had ≥2 infectious episodes. By severity, 27% of infections were CTCAE v5 grade three or higher and there were 2 sepsis-related deaths. Overall, flat-dose IVIG demonstrates feasibility for primary infection prophylaxis in RRMM patients receiving BsAbs.

## Introduction

Bispecific antibodies (BsAbs) are novel immunotherapeutic agents for relapsed/refractory multiple myeloma (RRMM) with prolific efficacy (1, 2). They have been associated with profound and prolonged hypogammaglobinemia which represents a unique challenge regarding increased risk of serious or fatal infections (3).

Historically, intravenous immunoglobulin (IVIG) has been dosed at 0.4 grams/kilogram for patients with multiple myeloma for infection prevention that has been based on prior data when lower potency chemotherapeutic agents were commonly used (4). Current expert recommendations are to administer IVIG to patients with MM who have hypogammaglobulinemia and recurrent infections (5).

There is still uncertainty in the optimal timing, schedule, and frequency of IVIG supplementation in patients with RRMM; especially in the setting of powerful immunosuppressing chemotherapeutic regimens, hematopoietic stem cell transplant (HSCT), and BsAbs all now commonly utilized for treatment (6). Recent data has shown a 90% lower rate of severe (grade 3-5) infections for patients with RRMM on anti-B-cell maturation antigen (BCMA) BsAb therapy when receiving weight-based IVIG dosing for prophylaxis compared to no infection prophylaxis at all (7, 8). Studies have also shown that IVIG prophylaxis reduces rates of subsequent infections more than than primary infection rates (9). Vanderbilt University Medical Center (VUMC) utilizes a uniform dose of 10 grams of IVIG for primary infection prophylaxis; an amount often equating to ~25%-33% of traditional weight-based dosing, depending on patient weight.

This study explores the impact of standardized IVIG dosing on infection rates in patients with RRMM treated with anti-G-protein coupled receptor class C group 5 member D (GPRC5D) and anti-BCMA BsAbs.

## Methods

### Patients and data collection

We performed a retrospective review of patients with RRMM who received either anti-BCMA or anti-GPRC5D BsAbs from January 2019 to December 2022 at VUMC, including both clinical trial and standard of care use. Inclusion criteria were a minimum of two months duration on BsAb therapy and the use of monthly IVIG prophylaxis at least once when serum IgG reached <400 mg/dL, regardless of prior infections. IVIG (Gammagard) was administered in 10-gram fixed doses monthly during routine MM disease monitoring when IgG level was <400 mg/dL, including in patients with IgG-based MM (in the absence of discernable assays). All patients were also on valacyclovir, trimethoprim-sulfamethoxazole, and levofloxacin for infection prophylaxis. Routine disease and treatment specifics as well as infection rates and grades were collected.

### Definitions

Myeloma disease staging was based on the revised international staging system (R-ISS). High-risk cytogenetics were defined by the presence of del17p, 1q21 gain/amplification, t(4;14), t(14;16), or t(14;20). Infections were graded based on Common Terminology Criteria for Adverse Events (CTCAE) v5 criteria. Neutropenia and lymphopenia were also graded based on CTCAE v5 criteria. Data was summarized with the use of descriptive statistics.

## Results

### Patient characteristics and disease history

A total of 30 patients with RRMM were identified. **Table 1** illustrates demographic and disease specifics. The median patient age was 70 (range 45-82) where 70% were male and 30% were female. There were 9 patients (21%) with extramedullary disease and 18 patients (43%) had >60% bone marrow plasmacytosis at the time of diagnosis. At receipt of BsAb, 27% of patients were R-ISS Stage I, 40% were Stage II, and 33% were Stage III. Most patients had a performance status score of 1 (83%) and the remainder had a score of 2 (17%). There were high-risk cytogenetics in 43% of all patients.

**Table 1:** Demographics, disease, and treatment characteristics of study patients.

Demographics	N (%) or Median (min-max)
Age	71 (45-82)
Men	21 (70%)
Women	9 (30%)
Caucasian	20 (67%)
African American	10 (33%)
Year of myeloma diagnosis	
2000-2010	7 (23%)
2010-2020	23 (77%)
Extramedullary Disease	6 (21%)
>60% bone marrow plasmacytosis at diagnosis	13 (43%)
R-ISS stage	
I	8 (27%)
II	12 (40%)
III	10 (33%)
ECOG performance status	
1	25 (83%)
2	5 (17%)
High Risk cytogenetics*	13 (43%)
Median Prior lines of therapy	4 (2-7)
Previous treatment exposure	
Triple Class	6 (20%)
Quad Class	9 (30%)
Penta Class	15 (50%)
Prior BCMA	9 (30%)
Refractory status to therapy	
Double Class	5 (17%)
Triple Class	8 (27%)
Quad Class	8 (27%)
Penta Class	2 (6%)
Previous ASCT	20 (67%)
Previous CAR-T (BCMA)	5 (17%)
Type of bispecific therapy	
Anti-BCMA BsAb**	12 (40%)
Anti-GPRC5D BsAb	18 (60%)
Duration of BsAb Therapy (months)	27 (12-45)

N, number; R-ISS, revised international staging system; ECOG, eastern cooperative oncology group; *, defined by the presence of del17p; 1q21, gain or amplification; t(4;14), t(14;16), t(14;20); BCMA, B cell maturation antigen; ASCT, autologous stem cell transplantation; CAR-T, chimeric antigen receptor T cell; **of these, 2 patients were treated standard of care, and rest received therapy on various clinical trials; GPRC5D, G protein-coupled receptor, class C, group 5, member D; BsAb, bispecific antibody.

The median number of lines of prior therapy was 4 (range 2-7) with 50% of patients exposed to penta-class agents. Of all patients, 27% were either triple-class or quad-class refractory and 6% were penta-class refractory. Prior autologous stem cell transplant occurred in 67% of all patients and 17% underwent prior treatment with BCMA-targeting CAR-T therapy.

Nearly all patients (93%) received BsAb therapy via clinical trial. Breaking down the type of BsAb received, 60% of patients received anti-GPRC5D BsAbs and 40% received anti-BCMA BsAbs. An anti-CD38 monoclonal antibody was added to BsAb therapy in twenty patients. Of these, two patients had an anti-BCMA BsAb backbone, and eighteen patients had an anti-GPRC5D BsAb backbone. The remaining ten patients received single agent anti-BCMA BsAb therapy. The two patients treated with BCMA BsAb with daratumumab did not experience significantly different infection-related adverse events compared with those receiving BCMA BsAb alone.

The median duration of BsAb-based therapy was 27 (range 12-45) months. The median time from BsAb treatment to IVIG commencement was 3.5 (range 0.13-12) months.

### Infectious episodes

**Table 2** illustrates the rates of infectious episodes and grades as well as IgG levels while on therapy. Overall, 73% of patients had at least one infection. Of those, 41% had only one infectious episode while 59% had two or more infections. The time to onset of first infection from therapy initiation was a median of 2 months (range 1-24). Twice as many patients had viral infections compared with bacterial infections (57% versus 30%). Overall, 27% of infections were CTCAE v5 grade three or higher. A majority of all infections (71%) were upper respiratory tract infections. There were 2 sepsis-related deaths: one of whom received anti-BCMA BsAb and the other received anti-GPRC5D BsAb.

**Table 2:** Infection, cytokine release syndrome, neurotoxicity, and non-hematologic complications on therapy and primary intravenous immunoglobulin prophylaxis.

	N (%) or Median (min-max)	Grade 1 N (%)	Grade 2 N (%)	Grade 3 N (%)	Grade 4 N (%)	Grade 5 N (%)
Number of infections						
1	9 (30%)					
≥2	13 (43%)					
Type of infection						
Bacterial	9 (30%)					
Viral	17 (57%)					
Severity of infections						
Mild	18 (60%)					
Moderate	7 (23%)					
Severe	2 (6.6%)					2 (6.6)
Upper Respiratory Infections			15 (50%)	4 (13%)	1 (3.3%)	
Infectious Diarrhea		4 (13%)				
Bacteremia				1 (3.3%)		
Sepsis						2 (6.6)
Urinary tract infection			1 (3.3%)			
IgG at start of BsAb Therapy	1284 (196-5431)					
IgG at First Infection	499 (210-2246)					
IgG at ≥ Second Infection	519 (110-1844)					
CRS	17 (57%)					
Grade 1	7 (23%)					
Grade 2	10 (33%)					
Time to onset of CRS (days)	3 (2-8)					
Duration of CRS (days)	2 (1-3)					
Neurotoxicity (NT)	3 (10%)					
Grade 1	3 (10%)					
Time to onset of NT (days)	9.5 (4-15)					
Duration of NT (days)	2 (1-3)					
Neutropenia at 1st infection		4 (13%)		2 (6.6%)		
Lymphopenia at 1st infection			6 (20%)	9 (30%)		
Non-Hematologic AEs						
Nail changes		4 (13%)	1 (3.3%)			
Skin rash		4 (13%)		2 (6.6%)		
Dysgeusia		9 (30%)				
Weight loss		5 (17%)	1 (3.3%)	3 (10%)		
Fatigue		3 (10%)	1 (3.3%)			

N, number; BsAb: bispecific antibody; CRS, cytokine release syndrome; AEs, adverse events.

The median IgG level was 1284 mg/dL (range 196-5431) at the time of diagnosis and 499 mg/dL (210-2246) at the onset of first infection. All patients except one received more than one dose of IVIG when IgG <400 mg/dL. The rate of grade three or higher neutropenia and lymphopenia was 6.6% and 30%, respectively.

## Discussion

Currently, weight-based dosing of IVIG is standard-of-care, and studies analyzing flat (limited, non-weight based) dosing of IVIG for infection prophylaxis have not been performed (6).

Our results demonstrate relatively low rates of grade ≥3 infections using a standardized 10g IVIG dosing schedule. Our cohort had 27% (n=8) of infections that were grade ≥3 compared to 44.8% and 7% of patients having grade 3 or 4 infections in the MajesTEC-1 and MonumenTAL-1 trials, respectively. Additionally, in our study, 73% of patients had at least one infection compared to 76.4% and 47% of patients in the MajesTEC-1 and MonumenTAL-1 trials, respectively.

Our study had relatively equal proportions of both anti-BCMA and anti-GPRC5D BsAb-treated patients (40% and 60%) and our data points fell in between the data of the clinical trial results above which is consistent with an average between the two studies. This is also consistent with recent evidence suggesting higher infection risk in patients treated with anti-BCMA BsAbs compared to anti-GPRC5D BsAbs (10). Some studies suggest that overall infection rates between the two agents are comparable but that anti-BCMA BsAbs have higher rates of severe infections, which may be due to the broader expression of BCMA on immune cells (11). Anti-BCMA BsAbs have higher rates of infection compared to anti-BCMA CAR-T therapy, partly due to the continued dosing of BsAbs (12).

Recent data found 41% of patients experienced grade 3–5 infections while receiving traditional weight-based IVIG dosing for infection prophylaxis in RRMM patients receiving anti-BCMA BsAbs (7). Real-world data looking at infection rates among patients treated with BsAbs found that 90% of patients had at least 1 infection and 41% had grade 3 or higher infections in a mixed cohort of both anti-BCMA and other BsAb-treated patients (13). Both studies demonstrate significantly higher grade 3 or higher infection rates than our study utilizing standardized IVIG dosing.

Previous data has shown that average IVIG doses for infection prophylaxis is approximately 38 grams with an average infusion time of 10 grams per hour and a rough IVIG cost estimate of $140/gram (14). If a standardized 10-gram IVIG dosing schedule were utilized, the average cost and time savings for each patient would be approximately $6,720 and 3 infusion hours monthly. As the demand for IVIG continues to increase with the expansion of BsAb therapies, reducing IVIG consumption is crucial in reducing cost (15).

Limitations of this study include its retrospective nature, relatively small sample size, and combination of both anti-GPRC5D and anti-BCMA BsAb-treated patients. In addition, nearly all patients were part of clinical trials, which may limit its applicability to real-world conditions. It is important to note that this analysis is exploratory in nature. We aim to study flat-based IVIG dosing further as this data demonstrates the feasibility of this potential approach with the aforementioned benefits. Future studies investigating differences in infection risks between anti-BCMA and anti-GPRC5D BsAb therapies would be helpful in determining if infection prophylaxis protocols should differ between the two agents. In addition, prospective validation with a randomized controlled trial comparing standardized IVIG dosing to traditional weight-based dosing will be essential.

This single institutional cohort of RRMM patients treated with anti-BCMA and anti-GPRC5D BsAbs demonstrates feasibility of a reduced, flat-dose IVIG protocol schedule for primary infection prophylaxis in RRMM and may help to conserve IVIG consumption and improve critical resource stewardship as BsAb therapies continue to expand and be used earlier in the treatment of MM.

## Data Availability

The original contributions presented in the study are included in the article/supplementary material. Further inquiries can be directed to the corresponding author.
